# Access to health services by women subjected to violence: findings from administrative healthcare data from the metropolitan area of northern Italy

**DOI:** 10.1186/s12905-025-03582-w

**Published:** 2025-02-12

**Authors:** Eliana Gabellini, Andrea Salvatori, Maria Teresa Greco, Cristina Cattaneo, Stefano Tambuzzi, Maria Antonella Costantino, Antonio Giampiero Russo

**Affiliations:** 1 Epidemiology Unit, Agency for Health Protection of the Metropolitan City of Milan, Milan, Italy; 2 Labanof-MUSA, Institute of Legal Medicine, Department of Biomedical Sciences for Health, University of Milan, Milan, Italy; 3 Institute of Legal Medicine, Department of Biomedical Sciences for Health, University of Milan, Milan, Italy; 4 Child and Adolescent Neuropsychiatry Service (UONPIA), Fondazione IRCCS Ca’ Granda Ospedale Maggiore Policlinico, Milan, Italy

**Keywords:** Women, Violence, Health services, Emergency department, Public health

## Abstract

**Background:**

In Italy, approximately 50% of women report experiencing episodes of psychological and/or physical violence. The Emergency Department (ED) is widely recognized as one of the health services to which victims of violence seek treatment for injuries and within which situations of domestic violence and abuse can be recognized. This work aims to estimate the phenomenon of violence in the population of the Health Protection Agency (ATS) of Milan using data from emergency room access and hospital admissions. A further goal of the survey is to provide evidence to launch audit processes in health units designed to foster improvement strategies for the management of the phenomenon and guarantee integration with anti-violence centers.

**Methods:**

In the first phase of the project, an algorithm was designed to detect women subjected to violence intercepted in the five years 2019–2023 in the administrative healthcare data of emergency rooms and hospital admissions. To identify the cases, the specific diagnosis codes of the ICD-9 have been selected. Prevalence and time trends of the phenomenon were estimated, stratifying by different socioanagraphic characteristics and by types and attributes of access.

In addition, the degree of integration between social and health services was analyzed. The areas researched concern intake by specialized services; specifically, data on female residents of the cohort were cross-referenced with data on access to the network of social health services in the ATS territory.

**Results:**

A clearly increasing phenomenon emerges: from 2019 to 2023, admissions to the ED have risen by 17%. A total of 35.6 percent of admissions involve women between the ages of 18 and 34. Foreigners account for approximately 40.9 percent of the cohort while constituting 15 percent of women residing in the territory. The yellow (Urgency) triage code is assigned for 3 out of the 4 admissions. A total of 11.5% of women had multiple accesses.

**Conclusions:**

Administrative healthcare data offer consistent data for studying the phenomenon of violence. This project aims to provide useful tools to better guide policies for intervention and integration between services in the area. Further reflection will focus on the possibility of integrating health and social services to support an integrated approach.

## Background

As the Council of Europe Convention on Preventing and Combating Violence against Women and Domestic Violence [14] states, the term “violence against women” is understood as a “violation of human rights and a form of discrimination against women and shall mean all acts of gender-based violence that result in, or are likely to result in, physical, sexual, psychological or economic harm or suffering to women, including threats of such acts, coercion or arbitrary deprivation of liberty, whether occurring in public or in private life”. According to the Italian Population Survey on Alcohol and Other Drugs study (IPSAD®), in Italy, nearly 12 million and 500 thousand (50.9%) women between the ages of 18 and 84 reported having been victims of psychological and/or physical violence at least once in their lifetime [13]. The WHO estimates that violence has devastating consequences from a physical and psychological perspective: it results in as serious a cause of death or disability for women of reproductive age as cancer and a more important cause of ill health than the effects of traffic accidents and malaria combined” [27, 29]. The consequences of violence are therefore profound and go far beyond the health of individual women, affecting the well-being of entire communities [18].

The Emergency Department (ED) is widely recognized as a health facility where people experiencing violence seek treatment for injuries and symptoms and in which they may disclose domestic violence and abuse situations. The ED setting frequently provides a point of reference for victims in the period before violence escalates to homicide or law enforcement intervention [12, 19]. For this reason, these services can play a strategic and essential role in interventions to prevent and combat violence, configuring themselves as suitable contexts for monitoring this issue.

To ensure adequate policies for preventing and combating gender-based violence in such settings, the “National Guidelines for Health Authorities and Hospital Authorities on Rescue and Socio-Health Care for Women Victims of Violence” were published in 2017 [16]. In this document, regulations are established to accelerate the speed of visits and ensure a decrease in waiting times in emergency rooms to minimize the risk of second thoughts, to implement a brief risk assessment procedure by health care professionals to assess the possibility of recidivism, to encourage integrated care, and to provide for the possibility, in the event of minor children, of a joint mother‒child pathway.

The Health Protection Agency (ATS) of the Metropolitan City of Milan covers the territory of 193 municipalities in the districts of Milan and Lodi. Over the past few years, coordination and dialog activities have been carried out in the territory, with local actors working to combat gender and domestic violence. All the municipalities that are part of the ATS basin are formally and actively involved in the eight networks that have been set up to contrast gender-based violence. Among the actions implemented was the creation of a working group with the referents of the 30 emergency rooms operating in the ATS territory with the aim of applying the aforementioned 2017 National Guidelines. This work has resulted in all the emergency services having guidelines that focus in particular on the reception of the victim, risk assessment, reporting, relations with the inter-institutional networks and with the anti-violence centres, as well as with the health and socio-health networks in the territory. With regard to the relationship with the territory, the legislation states that the health professionals who have taken charge of the woman must always inform her of the possibility of contacting the local anti-violence centres, the public and private services of the local network and, if the woman agrees, activate the territorial anti-violence network, alerting the actors involved in the formalised protocols.

This research aims to develop tools aimed at providing stakeholders with epidemiological data on the prevalence of women victims of violence accessing ATS Milan health facilities, given that few studies estimate the prevalence or incidence of the phenomenon using population-based data [7, 9]. The Report “Emergency Room Accesses of Women with Indications of Violence” (2023) from the National Institute of Statistics [10] was used as a reference. A specific in-depth study is carried out on the care of victims of violence by the health and social health services of the regional health system. Another objective of the work is to provide data and information to initiate audit trials in health facilities (particularly EDs) to launch care by the network of antiviolence centers, promoting improvement strategies for the management of the phenomenon.

## Methods

The study was conducted by querying the Administrative Healthcare Databases (AHDs) of the ATS of Milan, specifically, data from the ED and Hospital Discharge Sheets (HDSs) for the last 5 years (2019–2023). The spatial and temporal distributions of the phenomenon of violence were studied, taking into account the impact of the COVID-19 pandemic. To this end, accesses and admissions with diagnosis codes identified by the Report “Emergency Room Accesses of Women with Indications of Violence” (2023) of the National Institute of Statistics (Istat) and the Ministry of Health were researched. The emergency room admissions reporting mode 30 “other people's violence” in the variable “main problem” was also searched. Table 1 below shows the classification of the ICD-9-coded diagnoses studied.

**Table 1:** Violence diagnoses coded according to the ICD-9-CM classification

Codes	Types of violence
**Abused adult** (995.80 < = ICD-9-CM < = 995.85)	Emotional/psychological abuse of adult, Sexual abuse of adult, Neglect of adult (nutritional), Other.
**History of violence** (ICD-9-CM = V15.41, V15.42)	Personal history of psychic trauma from physical violence, Personal history of psychic trauma from emotional violence.
**Conjugal problems** (ICD-9-CM = V61.11, V61.12)	Abuse by spouse or partner, Abuse on spouse or partner.
**Observation for suspected violence** (ICD-9-CM = V71.5, V71.6, V71.81)	Observation following alleged rape or seduction, Observation following other voluntary injuries, Observation for suspected abuse and neglect.
**Injuries inflicted by other persons** (E960 < = ICD-9-CM < = E966, E968, E969)	Fighting, brawling, rape, Assault with a caustic or corrosive substance, poisoning, with hanging and strangulation, with firearms and explosives, with sharp and stinging instruments, Other, After-effects of injuries intentionally inflicted by other person.
**Abuser** (ICD-9-CM = E967)	Father, stepfather or boyfriend; mother, stepmother or girlfriend; spouse or partner; other relative (child, sibling, grandparent); other specified person (nonfamily member); caregiver; unspecified person.
**Specific diagnoses of minors**	
**Battered Child Syndrome** (ICD-9-CM = 995.5)	Emotional/psychological child abuse, Child neglect (nutritional), Child sexual abuse, Child physical abuse, and others.
**Parent‒child problems** (ICD-9-CM = V61.21, V61.22)	Child abuse, Abuse of the child by the parent.

In addition to data on the number of admissions to the health care system with a diagnosis of violence, the types and characteristics of admissions were examined: mode of arrival, triage code after medical examination, treatment outcome, and diagnoses related to violence. Ultimately, the issue of multiple access was addressed. Second, the sociodemographic and socioeconomic data of women victims of violence, such as age, citizenship, marital status, educational level, and deprivation index, were explored, with the awareness that gender-based violence is a pervasive phenomenon that cuts across different levels of income, class, and culture [28].

In addition to these elements, an investigation was carried out regarding the integration of social and health services that can help people who are victims of violence, with a focus on the connection between hospital services and territorial services. The areas researched involve territorial intake by specialized services (psychiatry, family counseling, outpatient clinics, etc.). Specifically, data on resident women in the cohort were cross-referenced with data on access to the network of social and health services in the ATS territory. These findings aimed to determine whether, following access to the ED, pathways have been activated at a territorial level to address the possible difficulties and consequences of violence for physical, mental, and social health. Methodologically speaking, we chose to consider only women living in the area between 2019 and 2023. Specifically, a cross-referencing procedure was carried out between the administrative data of Health and Social Care Outpatient Services (28-SAN), Family Counseling Services (CONS), and Territorial Psychiatry (46-SAN), checking for access within 12 months of entering health care services with an indication of violence. Specific services related to psychological support, psychotherapeutic interventions, self-help group meetings, and psychiatric visits were searched in the databases of outpatient and family counseling services.

## Results

### Women’s ED admissions and hospitalizations

Between 2019 and 2023, there were 11,589 admissions to the EDs of women victims of violence. There has been a notable increase in the last year: in 2023, there were 2365 women who accessed with an indication of violence in one of the ATSs of the Milan emergency rooms. Absolute frequencies have increased over time, excluding the lockdown due to the COVID-19 pandemic and the consequent general decrease in admissions to the hospital system.

The general growth is clearly manifested through the rates of total admissions to the ED. The rate of admissions with an indication of violence out of the total number of admissions (per 10,000) appeared to increase steadily and uninterruptedly during the peak of the pandemic period (2020–2021). It increased from 32.8 accesses in 2019 to 40.3 in 2020 and 39.6 in 2021, rising to 40.1 in 2022 and reaching 44.7 in 2023.

As part of shaping appropriate prevention and law enforcement policies, it is important to delve into the issue of multiple accesses of victims of violence. A total of 11.5% of women with admissions to the PS with an indication of violence had 2 or more accesses to different hospital departments in the territory in the five years 2019–2023 (Table 2).

**Table 2:** Information on access to health services

		**N**	**%**	**Admission rate per 10,000**
**N. of ED women**				
	**2019**	2103	21.1%	9.4
	**2020**	1564	15. 7%	7.1
	**2021**	1821	18.3%	8.3
	**2022**	2101	21.1%	9.4
	**2023**	2365	23.8%	10.7
**Total**^**a**^		9954	100.0%	
**N. of ED accesses**				
	**2019**	2414	20.8%	32.8
	**2020**	1836	15.8%	40.3
	**2021**	2090	18.0%	39.6
	**2022**	2436	21.0%	40.1
	**2023**	2813	24.3%	44.7
**Total**		11,589	100.0%	
**N. of multiple accesses**				
	**1**	8321	88.5%	
	**2**	859	9.1%	
	**3**	150	1.6%	
	**4**	49	0.5%	
	**5 and more**	23	0.3%	
**Total**^**b**^		9402	100.0%	
**Hospitalizations**^**c**^				
	**Yes**	441	4.7%	
	**No**	8961	95.3%	
**Total**		9402	100.0%	

^a^In this case, a single record per woman was maintained for each year

^b^Repeated accesses for any hospital per fiscal code were counted

^c^The number of women who had access to the emergency room due to violence and who were admitted to the hospital within 7 days of the access was studied

The diagnoses that were most commonly reported in ED admissions with an indication of violence corresponded to the ICD-9 codes indicated in the 2017 national guidelines mentioned above. A total of 41.5 percent of women in 2019–2023 were given the diagnosis “Unspecified Adult Maltreatment,” 15.7 percent were given the diagnosis related to “Adult Sexual Abuse,” and 12.1 percent were given “Battered Adult Syndrome.”

Most of the women arrived at the ED by their own means (56.4% of the total), while there has been an increase in access via ambulance over the past five years, rising from 6.2% in 2019 to 9.6% in 2023.

Over the five years, the yellow (Urgency) triage code was attributed to 3 out of 4 accesses. In this regard, there was an increase in the use of this code by 14.3% between 2019 and 2023. This shows that adherence to ministerial guidelines ensures that waiting times in emergency rooms are decreased to minimize the risk of second thoughts.

Approximately 87% of admissions from 2019–2023 ended with discharge to home. In the last year (2023), 83.3% of women who accessed were discharged, and 7 out of every 100 women abandoned the hospital facility (either during the course of investigation or before medical examination).

The total number of women hospitalized with an indication of violence was 388, for a total of 398 admissions. The number of hospitalizations decreased during the pandemic period and then increased in the previous year. The rates per 10,000 hospitalizations are expected to increase in 2023, with a statistic of 5 hospitalizations per 10,000. Women of foreign origin account for 55% of admissions.

The issue of hospitalizations was further explored by working on the merging of the emergency department database with the inpatient database to be able to more accurately determine how many emergency department admissions with violence were followed by hospitalizations, regardless of the diagnosis code reported for the inpatient.

Table 2 shows the results for the cohort of women who came to the Emergency Department with an indication of violence.

### Population characteristics

Approximately 9500 women accessed health facilities with an indication of violence between 2019 and 2023.

With respect to only female residents in ATS Milan (data on last place of residence), the data stratified by area show that the majority live in the province of Milan. According to the data by municipality, almost 4 out of 10 women live in the municipality of Milan. (see Table 3).

**Table 3:** Population characteristics

		**N**	**%**	**Admission rate per 10,000**
**Residence**^**a**^				
	Province of Milan	6241	90.6%	37.5
	*City of Milan*	*2601*	*37.76%*	36.5
	Province of Lodi	648	9.4%	56.4
**Total**		6889	100.0%	38.7
***Missing records***	*(nonresidents)*	*2638*		
**Age class (y.)**				
	0–17	1046	11.0%	39.5
	18–34	3308	34.7%	111.6
	35–49	3081	32.4%	83.1
	50–64	1555	16.3%	38.1
	65 +	535	5.6%	11.1
**Total**		9525	100.0%	52.3
***Missing records***		*2*		
**Area of birth**				
	Italy	4740	59.2%	31.7
	Other countries	3274	40.9%	100.5
**Total**		8014	100.0%	53.6
***Missing records***		*1513*		
**Area of birth – foreign women**^**b**^				
	Oceania	1	0.1%	
	North America	2	0.1%	
	Central-southern Africa	6	0.3%	
	West Asia	13	0.7%	
	East Africa	24	1.4%	
	West Africa	40	2.2%	
	Central-southern Asia	102	5.7%	
	Central and Eastern Europe	131	7.4%	
	East Asia	140	7.9%	
	North Africa	206	11.6%	
	European Union	539	30.3%	
	Central-southern America	578	32.4%	
**Total**		1782	100.0%	
***Missing records***		*1492*		

^a^It should be noted that approximately 27.5% of women who accessed health facilities with a diagnosis of violence did not live in the area of ATS Milan (Milan and Lodi provinces)

^b^The data collected belong only to those assisted by the health system of ATS Milan. Country-of-origin information was compiled for only about half of the foreign women cohort

In terms of age group, there were mostly women between 18 and 34 years old (34.7%), followed by those aged 35–49 years (32.4%) and those aged 50–64 years (16.3%). Underage women account for 11 percent of the total, whereas women over 65 account for 5.6 percent. (Table 3).

Looking at the data broken down by origin, foreign-born women account for 40.9 percent of the cohort. Overall, this represents a significant figure considering that foreign women represent approximately 15 percent of the resident population. Table 3 provides information on the area of origin of the foreign women in the cohort.

With respect to marital status, although there is no complete information, 6 out of 10 women reported being single/never married (61.5%). On the other hand, 32.9% of the women were married, whereas 5.6% were separated, divorced, or widowed at the time of access.

In terms of educational qualifications, 40.1% had a high school diploma, 37.4% had a middle school diploma, and 12.4% had a university degree. Finally, 7.5% had completed primary school, and 2.5% had no educational qualifications.

Regarding the deprivation index, women residing in the ATS area between 2019 and 2023 were considered, and data from the most recent census section were selected. The analysis revealed that 31.2% of women residing in ATS who entered healthcare facilities with a diagnosis of violence lived in deprived areas.

### Access to social and health services

A total of 21.7% percent of the resident women with access to or admissions to hospitals with an indication of violence made in the year following the event had at least one access to outpatient facilities, family counseling services, and territorial services for mental health care submitting demands and needs related to the dimension of psychological support.

Among the cohort of female residents with access to and hospitalizations for a diagnosis of violence, 12.4 percent used outpatient services in the 12 months following the event.

It appears that 7.4 percent of the women admitted to principals for violence had access to family counseling centers for issues related to mental health in the year following the event.

In the research carried out with the 46-SAN administrative healthcare data, the women's admissions within the mental healthcare facilities in the territory, which include Day Care Centers, PsychoSocial Centers, Residential Facilities (Protected Communities and Rehabilitation Communities) and Outpatient Clinics, were selected. It turns out that 6.5 percent of women with admissions to hospital facilities for violence were followed by one of the listed facilities in the 12 months following the event. However, it is worth mentioning that most of these people (i.e., 60.9%) were already in the care of territorial psychiatry in the year before the violent event.

## Discussion

According to the results of our study, women’s ED access with an indication of violence from 2019–2023 increased from 2414 to 2813. The overall increase in violence-related ED admissions in women is confirmed by the rate of ED admissions per year, which increased from 32.8 in 2019 to 44.7 in 2023. Data from the Italian Report “Emergency Room Accesses of Women with Indications of Violence” (2023) of the National Institute of Statistics (Istat, 2023) show the same trend, with values ranging from 14.1 in 2017 to 17.4 in 2022.

The international literature reports wide variability in this phenomenon, although data on trends in recent years are limited. Since our analysis is specific and addresses the phenomenon of violence in general, we do not have similar findings in international studies. Therefore, here, we discuss data on a specific phenomenon that has been the subject of various studies, namely Intimate Partner Violence (IPV). Intimate partner violence is one of the most common forms of violence against women and includes physical, sexual, and emotional abuse and controlling behavior by an intimate partner [30]. A recent systematic review reported that nearly 4 in 10 (37.3%) women aged 16 and over had experienced “any Intimate Partner Violence (IPV)” in their lifetime, and one in four women (24%) had experienced “any IPV” in the past year, confirming that the occurrence of violence is highly prevalent in global terms [35]. Another multicentric study conducted in most countries reported prevalence rates ranging from eight to 57% [33]. To analyze the extent of the phenomenon that comes to reporting, however, it is also useful to consider the data reported in an interesting Cochrane review. This study reports a range of women experiencing IPV ranging from 3 to 17%, with a median of only 8%, and pays attention to screening in healthcare settings. The authors reported that women seeking healthcare had a very low rate of IPV, and the results of the study show that screening increases the identification of women experiencing IPV in healthcare settings [31]. This shows that healthcare providers in the emergency department are in a privileged position where they can truly intercept cases of violence and intervene to ensure that the patient receives the right treatment and to prevent this from leading to fatal consequences for the woman in the most extreme cases, as can unfortunately happen [4].

The socio-demographic data are another aspect to consider. The results show that, in terms of age distribution, the largest proportion of women is in the 18–34 age group (34.7%) and that women under 18 represent 11% of the total. This highlights the significant presence of young people under the age of 35 who are subjected to violence, and it is consistent with evidence at European level that shows increasing concern about the extent of IPV among young women [32].

In terms of socio-economic characteristics, 31.2 percent of women subjected to violence live in deprived areas. These data were calculated using the Deprivation index, a multidimensional measure of relative deprivation, both material and social, computed at the level of geographical aggregates that can be used as a proxy for the level of individual social deprivation [25]. The proportion of women living in deprived areas falls to 24% when looking at the total number of females living in the ATS territory: this suggests a higher prevalence of women in such disadvantaged conditions in the study cohort.

The basic assumption is that gender-based violence intersects all classes. However, there are a number of relevant studies to be taken into consideration that highlight the complex interaction between socio-economic factors and this phenomenon. A report prepared at the request of the European Commission [5] on how a woman's economic independence and the wider economic conditions that she, her partner, and her family face affect violence that provides a detailed picture of the issue. The empirical research presented in this report using the recently published FRA Survey data on violence against women shows that, in terms of physical violence, household poverty is positively and significantly associated with the risk of physical abuse. With regard to sexual violence, its prevalence rates suggest that women living in more deprived households are at higher risk of sexual violence than women in other types of households, particularly those living in wealthier conditions. However, the estimated probabilities of sexual abuse suggest that the differences between families' economic conditions are not robust enough to reach statistical significance.

Another risk factor, again in the area of domestic violence, relates to the characteristics of perpetrators, as it has been found that there are a number of vulnerabilities created by male unemployment that can increase the risk of violence for their partners. Other studies, however, on domestic violence, point out that separation, being a single mother, debts of a partner or ex-partner, and the barriers violence creates to employment, are all factors that may be the consequence of violence and plunge women into poverty, rather than poverty itself being the risk factor for violence [20].

Other studies have also investigated the influence of ecological factors and socio-economic inequalities on violence. Contextual factors, such as socioeconomic inequity and area-level disadvantage, have been associated with gender-based violence, and these factors likely interact with the built environment and gender inequality to shape the spatial distribution of gender violence [2].

Given that the focus of this article is on access to health services with an indication of violence, it is crucial to emphasize the intersection between socioeconomic disparities and the patterns or types of violence experienced. Since violence-related ED visits with an indication of violence in the ATS area are mainly related to situations of physical and sexual abuse, the data presented on women in socio-economically disadvantaged conditions are consistent with the evidence presented above, especially at the European level.

In addition to the issue that has just been discussed, there is another piece of evidence that is of particular interest. The high attendance of women with migratory backgrounds is a significant element. ED admissions of women with a migrant background account for about 41.7% of the total, although they make up about 15% of the women resident in the area. Moreover, foreign women account for the majority of hospitalizations: over the five-year period, this group constitued 55% of admissions. A study conducted in Italy on AHD of two large regions revealed that being foreigner increases the probability of being victim of violence in both children and women [24], and another research on ED database of Lazio Region showed that the prevalence of ED visits for violent episodes in women is higher among young and foreign women [7, 9].

There are various studies in the literature on the phenomenon of violence against foreign-born women, and there are several elements to be taken into account. On the one hand, it is crucial to remember the difficulties that migrant women face on their way to Italy: many women are forced to pay for their migration through prostitution or are subjected to brutal sexual exploitation and torture on the journey, particularly in the Central Mediterranean route [3]. A systematic review [8] aimed at documenting the violence experienced by immigrant women in their host country and its prevalence found that, although the migrant population is heterogeneous (coming from different countries of origin and having many different reasons for leaving their home countries), there are certain common characteristics and barriers they face in their host countries that may contribute to increasing their vulnerability to different types/forms of victimization. For example, some of these women immigrated illegally, some are confronted with cultural differences and alienation due to social isolation resulting from the migration process; others face a lack of social support and experience social exclusion, poverty, and economic dependency. Furthermore, the immigration policies of host countries can themselves increase women's vulnerability to violence, especially for undocumented women, which brings them to the services in the most extreme situations. Beyond this aspect, it is also crucial to reflect on the use of social and health services, specifically the ED Department, by foreign users, although access patterns differ according to origin and context of arrival [1]. In a systematic review conducted in Europe [6], key findings in the Italian context include a greater propensity to use ED by the population with a migrant background compared to the Italian population. With regard to Italian legislation, it is important to remember that resident immigrants have the same right to access and use health services as the Italian population, whereas undocumented immigrants are only covered for emergency care and preventive care and treatment related to communicable diseases and pregnancy and childbirth. Factors explaining the overuse of emergency services by native-born immigrants include: an easier response to the health needs of immigrants, which provides an immediate solution to a given health problem; language difficulties; and a lack of knowledge of the social and health service system, which may lead to emergency services being used as a substitute for existing services [15].

The data presented, as well as elements from the literature regarding this issue, need to be considered when constructing future policies that take into account cultural diversity.

According to the Italian study on the data on calls from users of the 1522 telephone number (Italian Anti-Violence and Stalking Number) and victims of violence who turn to this service for help, three quarters of the victims who turn to the helpline do not report the violence they have suffered to the competent authorities (70.9%) and the reasons for not reporting are mainly fear, for themselves and of the perpetrator's reaction [22]. With respect to screening, according to the 2014 EU Violence Against Women Survey 67% of women in the EU who experienced physical or sexual violence did not report the most serious incident of partner violence to the police or other organisations [17]. In recent years, the Italian Ministry of Health has promoted projects to increase the awareness of social and health workers who encounter victims to identify signs of violence [21], which may have resulted in an overall increase in the prevalence of the phenomenon. However, raising awareness among healthcare staff is only the first step, as they should be trained to be prepared for different situations, since each type of violence has its own causes, risk factors and health consequences. Therefore, there is an urgent need to train ED physicians in how to conduct a clinical forensic evaluation (starting from anamnesis) and appropriate legal procedures for victims of interpersonal violence to promote social justice and assist communities in sentencing offenders [34]. In fact, the optimal solution could be the actual presence of forensic doctors and forensic nurses in emergency departments, as the reform of legal medicine in France of January 15, 2011, has clearly shown [11]. In fact, physicians in emergency departments are often busy with other urgent clinical activities and do not have time to properly investigate cases of violence, even if they are trained to do so, as a research study at the Policlinico Hospital of Milan noted [34]. Therefore, many cases are not approached correctly from the outset and it is worth pointing out that in the current study, a significant number of the accesses with an indication of violence were not coded with the codes provided by the Italian guidelines. In fact, a significant number of ED admissions were identified solely by the information contained in the section on the Main Problem.

In addition, several studies have highlighted the importance of adapting screening tools to consider specific issues [26], especially for vulnerable groups such as people with disabilities, the elderly, and foreigners. The work of Messing et al. [23] on this topic showed that the adapted version of the Danger Assessment for Immigrant Women predicts the risk of IPV victimisation more accurately than the original tool, as it takes into account risk factors specific to the immigration context.

With respect to the analysis covered in our study of the use of health and social services oriented toward psychosocial support, at least one in five women gained access to the offer present in the territory following the episode of violence. One element of interest that could not be investigated concerns the networking between hospitals that intercept women and antiviolence centers because the data of women accessing the anti-violence network are protected by specific regulations and are therefore not available in health and social health databases.

Several aspects can be highlighted regarding the work of the Lombardy healthcare system in relation to the response and the support network for women victims of violence. The anti-violence system of the Lombardy Region is based on the presence of 27 territorial inter-institutional networks that include: anti-violence centres, emergency rooms, family counselling centres, law enforcement agencies, the judiciary, social services, the third sector and shelters. In its health protection mandate, the ATS is committed to ensuring the management of networks and coordination with the services of the local health authorities, in particular the emergency departments. Anti-violence centres can work in synergy with health and hospital structures and can be a reference point for and support to the emergency services, following the establishment of specific agreements/conventions. With decree No. 9146 of 17 June 2024, the Lombardy Region approved a call for tenders addressed to all social-health agencies in the Lombardy region for the identification of experimentations at territorial level for the integrated taking charge of women victims of violence with or without children. As a result, several projects have been approved with the following areas of work: integration with local social networks, 24-h emergency services, specialisation courses for operators and integrated care of minors who are victims of witnessing violence.

## Conclusions

This study provides a comprehensive and detailed overview of the phenomenon of violence against women intercepted by Health Care Providers in the territory of ATS Milan. This study represents significant information heritage, and it is important as a starting point for reflection on the trend of the phenomenon and on the capacity of Health Care Providers to act and recognize situations of violence. In this regard, the evidence shown can be used to advocate for multilayered, systems-based approaches to strengthen health responses to violence against women. In this context, with this information, audit trials can be initiated with healthcare facilities to work on the awareness and training of practitioners while considering the data for individual territorial articulations/facilities. Clinical forensic medicine can make important contributions to all of this.

The limitations of the study are the lack of integration of health data and social and antiviolence center data. Sharing this information would make it possible to assess the ability of healthcare providers to put the person in contact with specialized services, thus ensuring that the network of anti-violence centers takes charge. At present, however, reducing the number of unreported cases of women who are victims of domestic violence should be the primary goal that society and healthcare professionals should set themselves.

In the future, it would be necessary to study outreach and integration between services in the territory, paying attention to more fragile and vulnerable individuals, such as women with migratory backgrounds, women with mental health problems, and women with disabilities.

## Data Availability

The dataset generated and analysed during the current study is not publicly available due to privacy concerns, as it originates from administrative healthcare databases, which are subject to privacy restrictions.

## Abbreviations

ATS of Milan: Agency for Health Protection of the Metropolitan City of Milan
ED: Emergency Department
IPV: Intimate Partner Violence
AHD: Administrative Healthcare Databases
HDS: Hospital Discharge Sheets
